# Artificial intelligence-enabled atrial fibrillation detection using smartwatches: current status and future perspectives

**DOI:** 10.3389/fcvm.2024.1432876

**Published:** 2024-07-15

**Authors:** Zoi Papalamprakopoulou, Dimitrios Stavropoulos, Serafeim Moustakidis, Dimitrios Avgerinos, Michael Efremidis, Polydoros N. Kampaktsis

**Affiliations:** ^1^ Department of Medicine, Division of Gastroenterology, Hepatology and Nutrition, University at Buffalo School of Medicine and Biomedical Sciences, Buffalo, NY, United States; ^2^ Department of Ophthalmology, University of Pittsburgh School of Medicine, Pittsburgh, PA, United States; ^3^ AiDEAS, Tallinn, Estonia; ^4^ Onassis Cardiac Surgery Center, Athens, Greece; ^5^ Department of Medicine, Aristotle University of Thessaloniki Medical School, Thessaloniki, Greece

**Keywords:** atrial fibrillation, smartwatches, photoplethysmography, single-lead electrocardiography, artificial intelligence, machine learning

## Abstract

Atrial fibrillation (AF) significantly increases the risk of stroke and heart failure, but is frequently asymptomatic and intermittent; therefore, its timely diagnosis poses challenges. Early detection in selected patients may aid in stroke prevention and mitigate structural heart complications through prompt intervention. Smartwatches, coupled with powerful artificial intelligence (AI)-enabled algorithms, offer a promising tool for early detection due to their widespread use, easiness of use, and potential cost-effectiveness. Commercially available smartwatches have gained clearance from the FDA to detect AF and are becoming increasingly popular. Despite their promise, the evolving landscape of AI-enabled smartwatch-based AF detection raises questions about the clinical value of this technology. Following the ongoing digital transformation of healthcare, clinicians should familiarize themselves with how AI-enabled smartwatches function in AF detection and navigate their role in clinical settings to deliver optimal patient care. In this review, we provide a concise overview of the characteristics of AI-enabled smartwatch algorithms, their diagnostic performance, clinical value, limitations, and discuss future perspectives in AF diagnosis.

## Introduction

Atrial fibrillation (AF) constitutes the most common cardiac arrhythmia with a prevalence that is increasing. According to the Global Burden of Diseases study, the number of individuals affected by AF has doubled from 1990 to 2019, reaching approximately 60 million globally (1). This upward trajectory is projected to continue, with estimates suggesting that by 2030, the United States alone could see over 12.1 million people diagnosed with AF (2).

AF is associated with a fivefold increased risk of stroke, as well as an increased risk for heart failure (3, 4). However, its episodic manifestation and frequently asymptomatic nature, pose significant challenges for AF diagnosis (5). For instance, research data suggest that paroxysmal AF can be detected in 10%–20% of patients with cryptogenic strokes and extended monitoring with implantable devices is recommended in this population to increase sensitivity of AF detection (6–8). In selected asymptomatic patients, early AF detection could be helpful in preventing stroke using oral anticoagulation (OAC) or preventing heart failure using rhythm control strategies (4, 9).

Smartwatches empowered by artificial intelligence (AI) algorithms have emerged as a promising tool for early detection of AF (10, 11). Recent cost-effectiveness studies also underscore their potential to serve as a widespread tool for early arrhythmic detection (12). However, AF confirmation still requires electrocardiography (ECG) and widespread incorporation of smartwatches for AF detection has not been established (13–15).

In this review we first summarize the characteristics and performance of smartwatches that use AI-enabled algorithms for AF detection; secondly, we discuss their clinical applicability, the challenges around their use, and finally outline future directions in the field.

### Smartwatch technology for AF detection

#### Photoplethysmography

A common technology for detecting AF with smartwatches is photoplethysmography (PPG). This technique involves illuminating the skin with a light-emitting diode (LED) and detecting the light reflected back with a photodetector (16). The intensity of the reflected light constitutes the PPG signal, which varies according to blood volume changes in the vessels throughout the cardiac cycle. Therefore, the PPG signal represents a pulse pressure waveform that enables passive, continuous, or semi-continuous heart rhythm monitoring measured at the wrist (17).

Since various neural, cardiac, and respiratory factors regulate blood flow, physiologic cardiovascular parameters such as heart rate, blood pressure, oxygen saturation, and respiratory rate could be derived from PPG signal analysis (18). Importantly, the peak of the PPG signal correlates closely with the R wave of the electrocardiogram (ECG) (19). As such, the PPG signal can provide R-R intervals, i.e., heart rate variability (20) and in AF, the PPG signal exhibits greater irregularity of pulse waveform (16). Assessment of PPG variability through a detection algorithm is used to detect AF.

#### Single-lead ECG

Another common method for detecting AF with smartwatches is based on the recording of a single-lead ECG. In this method, the watch's back acts as a positive electrode and the contralateral fingertip is placed on the crown, acting as a negative electrode (21). This creates a bipolar ECG-lead, simulating Einthoven's ECG lead I. The recorded ECG tracing is typically saved and can be analyzed either manually by physicians or automatically. In addition to single-lead ECG, other techniques further explore the use of a smartwatch-based wireless 6-lead limb-like ECG, which requires interpretation by cardiologists, combined with PPG to increase accuracy of AF detection (22).

#### Detection algorithms and machine learning

Detection algorithms may rely on traditional statistics or AI, particularly machine learning (ML) and deep learning (DL) algorithms (23). For commercially available smartwatches, the AI-empowered algorithms utilized for AF detection remain proprietary to each company (24, 25). Nonetheless, we will provide a brief overview of how similar algorithms described in the research setting function to aid physicians in understanding their mechanisms. PPG or ECG signals, once captured, undergo several preprocessing steps including noise reduction, normalization, and segmentation. Feature extraction focuses on both time-domain and frequency-domain attributes, which might include heart rate variability indices, the morphology of the PPG or ECG waveform, and temporal intervals between heartbeats. A tachogram, a visual representation of heart rate variability plotted against time, serves as a key component in the PPG-based AF detection process (26). Typically, a set number of irregular tachograms over a period of time is involved in the algorithm's decision to trigger an irregular pulse notification (IPN). ML models such as support vector machines and random forests analyze these engineered features to classify heart rhythms, benefitting from the clear delineation of feature-based input (27, 28). In contrast, DL models such as convolutional neural networks (CNNs) and recurrent neural networks (RNNs) can process raw or minimally preprocessed signal data (29, 30). These models are adept at automatically detecting complex patterns within large datasets, which is beneficial for identifying subtle and non-linear indicators of AF. CNNs, for instance, are useful for spatial pattern recognition within ECG signals, while RNNs excel at analyzing sequential data, capturing dynamic changes over time which are crucial for continuous ECG monitoring (31).

#### Challenges

PPG sensors can yield missing signals or inconclusive/unclassified rhythms. Missing signals are often due to poor sensor-skin contact. On the other hand, insufficient quality of the signal may be attributed to motion artifacts, such as from muscular motion. Overall, PPG sensors tend to underestimate AF detection at both higher and lower heart rates when compared to standard detection methods (32). Additionally, ectopic beats can further complicate AF detection by generating pulse irregularities (22). Although evidence remain inconclusive, skin pigmentation may also restrict PPG performance (33, 34). Moreover, the PPG signal may be weaker in elderly, due to pathologic or physiologic changes associated with aging including reduced peripheral blood flow, increased arterial wall stiffness, and skin changes (35, 36). On the other hand, there may be a low amplitude of P-waves with single-lead ECG, which challenges the classification of electrical activity (37). Lastly, limitations of using smartwatch to detect AF include the necessity for user cooperation and regular charging, depending on its battery life (10).

Similarly, AI models, despite their capabilities, require robust, diverse datasets for training to ensure effective performance across various demographic groups and conditions. Additionally, these models necessitate significant computational resources, particularly in DL frameworks, to process extensive data and learn through sophisticated layers of abstraction. The interpretability of these AI systems is also crucial, particularly in healthcare settings, where providers prefer algorithms that offer insights into their decision-making processes (38). This aligns with the increasing demand for explainable AI in clinical environments, highlighting another significant limitation.

### Diagnostic performance of Ai-empowered smartwatches in AF detection

Currently, AF diagnosis requires that a physician reviews a standard 12-lead ECG or rhythm strips (9, 39). This also serves as the gold standard to determine the diagnostic accuracy of smartwatches. Table 1 summarizes key recent studies conducted from 2019 and onwards that evaluated the diagnostic performance of smartwatches for detecting AF.

**Table 1:** Key studies that evaluated the performance of smartwatches for AF detection.

Authors (year)	Study type	Aim of the study	Number of participants (*N*)	Study population	Smartwatch	Sensor/confirmatory test	Reference standard	Incidence & diagnostic performance
Perez et al. (40)	Prospective, siteless, pragmatic, population study	Screen for AF with IPN and confirm diagnosis with continuous ECG monitoring	419,297	≥22 yo with a compatible Apple iPhone and Apple Watch 6% were aged ≥65 yo Exclusions: known AF, OACs	Apple Watch Series 1–3	PPG	Single-lead ECG patch monitoring ×1 week in those with IPN only (clinician-interpreted)	IP*N* = 0.52% PPV = 84.0%
Lubitz et al. (41)	Prospective, siteless, pragmatic, population study	Screen for AF with IPN and confirm diagnosis with continuous ECG monitoring	455,699	≥22 yo with a compatible Fitbit wrist-worn device and Android/iOS smartphones 12% were aged ≥65 yo Exclusions: known AF, OACs, pacemaker/defibrillator	Fitbit wrist-worn device	PPG	Single-lead ECG patch monitoring ×1 week in those with IPN only (clinician- interpreted)	IPN = 1.0% PPV = 98.2% SN = 67.6% SP = 98.4%
Badertscher et al. (43)	Prospective, observational, single-center study	Assess the diagnostic accuracy of a novel smart device in detecting AF in cardiac patients	319	Patients who presented to the cardiology department Median = 67 yo; interquartile range 54–76 yo.	Withings Scanwatch	Single-lead iECG	12-lead ECG (cardiologist-interpreted)	SN = 76.0% SP = 99.0% *κ* = 0.72
Bacevicius et al. (22)	Single-center, non-randomized validation study with a prospective case-control model	Assess the diagnostic accuracy of a novel system in detecting AF when challenged by PVCs/PACs	344 (AF: *n* = 121)	≥18 yo, known AF, SR, or SR with frequent PVCs/PACs Exclusions: regular pulse wave despite AF, other arrhythmia	Wrist-worn device	PPG/on-demand watch 6-lead ECG	ECG Holter monitor (cardiologist- interpreted)	PPG + 6-lead ECG for the PVCs/PACs group: SN = 94.2%, SP = 99.6%, Accuracy = 99.5%
Avram et al. (42)	Prospective remote-based study	Estimate the SN and SP of a smartwatch for continuous detection of AF and calculate AF burden	204	Known AF or at risk for AF (age ≥ 65 yo, history of hypertension, diabetes, heart failure, or coronary disease) 48.0% were aged ≥65 yo	Samsung Galaxy Watch Active 2	PPG/on-demand watch single-lead iECG	Continuous 28-day ECG patch monitor (cardiologist and technician- interpreted)	PPG:SN = 87.8% SP = 97.4% PPG + single-lead ECG: SN = 96.9 SP = 99.3
Seshadri et al. (44)	Prospective	Assess the diagnostic accuracy of a smartwatch in detecting AF in cardiac patients	50	Patients who had undergone cardiac surgery and were on telemetry Exclusions: pacemaker, surgical use of a radial artery, chronic HR abnormalities, wrist tattoos	Apple Watch Series 4 (AW4) that used a notification and a downloadable waveform on PDF	Single-lead iECG	Telemetry	AW4 notification: SN = 41.0% SP = 100.0%
Rajakariar et al. (45)	Prospective, multi-center, validation study	Evaluate the accuracy of a novel smartwatch-based ECG monitor for diagnosing AF in an unselected high-risk inpatient population	200 patients (AF: *n* = 38)	Consecutive patients ≥18 yo admitted to the medical, cardiac or intensive care ward Exclusions: cardiac implantable devices, unable to independently use the device, or in contact isolation	AliveCor KardiaBand smartwatch-based HR monitor	Single-lead iECG	12-lead ECG (cardiologist- interpreted)	SN = 94.4% SP = 81.9% PPV = 54.8% NNP = 98.4% κ = 0.60
Mannhart et al. (46)	Prospective, single-center, diagnostic study	Assess the accuracy of 5 smart devices in identifying AF	201 patients (AF: *n* = 62)	≥18 yo scheduled for catheter ablation procedures, electric cardioversions, pacemaker, or ICD implantation Exclusions: 3 or less single-lead ECG recordings due to logistical or technical issues or missing data.	Apple Watch 6, AliveCor KardiaMobile or KardiaMobile 6l, Fitbit Sense, Samsung Galaxy Watch 3, and Withings Scanwatch ECG	Single-lead iECG	12-lead ECG (physician-interpreted)	Apple Watch: SN = 85.0%, SP = 75.0% Samsung Galaxy Watch 3: SN = 85.0% SP = 75.0% Withings Scanwatch: SN = 58.0% SP = 75.0% Fitbit Sense: SN = 66.0% SP = 79.0% AliveCor KardiaMobile: SN = 79.0% SP = 69.0%

AF, atrial fibrillation; IPN, irregular pulse notification; ECG, electrocardiogram, yo, years old; iECG, intelligent electrocardiogram; OACs, oral anticoagulants; PPG, photoplethysmography; PPV, positive predictive value; SN, sensitivity; SP, specificity; CI, confidence interval; κ, kappa coefficient; HR, heart rhythm; PVCs, premature ventricular contractions; PACs, premature atrial contractions; SR, sinus rhythm; ICD, implantable cardioverter-defibrillator; TIA, transient ischemic attack.

Large-scale, pragmatic studies that were conducted for major smartwatch companies like Apple and Fitbit assessed the ability to screen for AF in the general population relying on PPG (40, 41). The Apple Heart Study evaluated over 400,000 individuals (mean age 41 ± 13 years, 42% women) for IPN with the Apple Watch indicating possible AF in that event (40). The duration of PGG monitoring and the criteria to triggering an IPN for AF varied significantly among studies assessing PPG sensors. PPG monitoring was more intermittent with the Apple algorithm, where the IPN required at least 5/6 consecutive irregular 1-minute tachograms within a 48-hour period (40). Conversely, the Fitbit algorithm allowed for more continuous monitoring and applied stricter notification criteria, requiring at least 30 min of irregular rhythm before notifying the user (41). Participants who received such an IPN notification received a single-lead ECG patch via mail to wear for 1 week, which was used to diagnose AF. Overall, only 0.52% of the study population received an IPN using the Apple algorithm, with the percentage correlating with increased age (≥65 years: IPN 3.2%). Similarly, the Fitbit Heart Study assessed the performance of the Fitbit Watch among over 400,000 participants (median age 47 years, interquartile range 35–58 years, 71% women), of whom 1% received an IPN (≥65 years: IPN 3.6%) (41). Of those who received an IPN in the Fitbit and Apple Heart studies, only up to 25% of all notified participants in these studies returned the ECG patch. 32.2% and 34.0%, respectively were confirmed to have AF lasting at least 30 s on the reference ECG patch. The positive predictive value (PPV) for AF, confirmed concurrently on the ECG patch, of the Apple algorithm was 84.0% (95% CI, 76.0%–92.0%). The PPV was lower among those over 65, i.e., 78% (95% CI, 64.0%–92.0%). The Fitbit algorithm yielded a sensitivity of 67.6%, specificity of 98.4%, and PPV of 98.2% (95% CI, 95.5%–99.5%), with a slight reduction among those aged ≥65 years at 97.0% (95% CI, 91.4%–99.4%).

Among studies conducted in research settings and among populations at high-risk for AF, the performance of PPG sensors varied with sensitivity ranging from 87.8% (42) to 94.2% (22) and specificity up to 99.1% (22). Other studies examined the performance of intelligent ECG (iECG), a smartwatch-based single-lead ECG with automatic AF detection function (43–46). AF was considered present if episodes lasted for at least 30 s. The sensitivity of automatic AF detection with iECG varied from as low as 41.0% among post-cardiac surgery patients (44) to 94.4% among high-risk inpatient populations (45), while the specificity ranged from 69.0% (46) to 100.0% (44). These performance indices should be interpreted with caution, as most of these studies employed intention-to-diagnose analyses, excluding a significant number of tracings that were deemed inconclusive. Badertscher et al. demonstrated a reduction in such inconclusive tracings from 14% to 4.1% after a cardiologist's review, and this was further supported by Mannhart et al. (43, 46). Avram et al. demonstrated that obtaining a confirmatory iECG one hour apart the first one improved the specificity (100.0%) without affecting sensitivity (96.0%). Moreover, novel AI algorithms have demonstrated excellent performance in reducing inconclusive classification rates (47). Lastly, in most studies, patients were instructed on how to use the technology for AF detection which may not reflect real-life circumstances or digital literacy, especially among the elderly.

### Clinical impact of AF detection using Ai-empowered smartwatches

#### Potential benefits

The main potential advantage of detecting asymptomatic AF using smartwatches lies in the opportunity for early diagnosis and therapeutic intervention. Identifying AF among high risk, asymptomatic patients could lead to the initiation of OAC, potentially reducing the risk for stroke (4). The eBRAVE-AF trial showed that PPG-based screening more than doubled the detection rate of asymptomatic AF leading to subsequent OAC initiation (48). A recent meta-analysis demonstrated a significant reduction in stroke risk after OAC initiation in patients with asymptomatic AF detected by implantable cardiac devices (49). However, the results of studies investigating the specific clinical benefits of smartwatch-detected AF and the populations to which may apply, are pending (50). Additionally, increasing evidence suggests that early initiation of rhythm control not only lowers the risk of stroke, but also reduces the structural, long-term complications of AF, particularly HF (51, 52). Furthermore, identifying AF early could prompt the early identification and management of other concomitant cardiovascular conditions such as hypertension, underlying heart disease and diabetes mellitus (53). Lastly, population screening for AF using smartwatches is proposed as a more cost-effective approach compared to no screening or conventional screening (12). Nevertheless, no consensus has been reached yet regarding AF screening due to a lack of clinical data to support its potential benefits in asymptomatic patient groups at risk for AF and its complications (5, 9, 54).

#### Identifying populations for smartwatch-based AF detection

In the absence of relevant guidelines, it may be reasonable to introduce smartwatch AF screening in certain populations. For example, the 2020 ESC Guidelines for AF recommend AF screening for patients over 75 (5). Additionally, the same guidelines suggest screening for those at high stroke risk, though the optimal cut-offs on assessment tools, e.g., CHA2DS2-VASc score, for smartwatch-based AF detection require further clarification. Oppportunistic screening for AF is also suggested for patients aged 65 years and above, where smartwatches have already been incorporated as screening options (5). In contrast, the US Preventive Services Task Force implies that the current evidence is insufficient to assess the balance of benefits and harms for AF screening (54). It is noteworthy that AI algorithms utilizing Electronic Medical Records (PULsE-AI, FIND-AF) or ECGs (BEAGLE) may facilitate the identification of high-risk patients (55–57). However, it would be difficult to envision that smartwatches could be used for screening or substitute other established methods of AF detection in symptomatic patients at high risk for AF, such as those with cryptogenic stroke.

#### Clinical challenges and risks

Once the smartwatch indicates possible AF, the main question is how healthcare providers should proceed (10). In existing clinical studies, individuals were referred to contact a healthcare provider (40, 41, 58). Healthcare providers then confirmed the presence of AF through a standard ECG confirmatory test. This approach aligns with the 2020 ESC Guidelines for AF, which included smartwatches in the screening recommendations (5). However, the introduction of smartwatches for AF diagnosis poses risks for putting high stress on the healthcare system, particularly if extended population screening is implemented (59, 60). Therefore, it is crucial to optimize the parameters of smartwatch algorithms to minimize false positives and prevent physician overload. A study conducted among patients with known AF who utilized wearables, demonstrated increased engagement in follow-up healthcare, with no significant difference in pulse rates compared to those not using wearables (61). Further research into health outcomes will guide management for both patients and providers.

#### Data management

Smartwatch data management poses an ongoing challenge. The FDA does not classify smartwatches as medical devices; rather they are considered wellness tools, subject to expedited regulatory approval through the Digital Health Software Precertification (Pre-Cert) Program (62). This regulatory framework promotes the development of a large digital health market and the generation of a substantial volume of digital data (21). Despite this, smartwatch data have not yet been fully integrated into clinical workflows. While meaningful integration of smartwatch data into the existing Electronic Health Records (EHRs) will eventually become necessary, the specific type and volume of data to be shared with EHRs is yet to be determined. Consequently, there is an ongoing effort to endorse partnerships between startup organizations that specialize in integrating such data into EHRs with healthcare systems and insurance companies (63). It is also crucial to develop clear legislation regarding data ownership, ensure compliance with HIPAA regulations and address patient confidentiality, privacy, and security concerns (59, 64). Furthermore, attention is required concerning physician reimbursement, including the creation of standard documentation processes and billable codes related to the integration of smartwatch data into clinical workflows (65). With all these impending changes, it has been suggested that a “digital health counselor” could provide support and guide patients and providers throughout the initial transition phase of digital data integration into clinical workflows (59).

## Conclusions and future directions

AI-empowered smartwatches have emerged as a potential screening tool for AF, the most common arrhythmia with an increasing prevalence. Potentially important advantages of this strategy include purely non-invasive nature, easiness of use, early detection of AF in high-risk asymptomatic individuals and reduction in AF burden. However, there is no consensus among pertinent societies regarding AF screening and the clinical impact of this strategy using smartwatches remains to be proven. Particular issues that have to be addressed are: identification of populations that could benefit from AF screening using smartwatches; optimization of AI algorithms to improve diagnostic accuracy and reduce confirmatory tests; data management of protected health care information including infrastructure for processing of large amounts of data, and clinical algorithms to assist with widespread application of AF screening via smartwatches. Large trials to address these questions are currently required and may expect the landscape of AF in the near future.
